# Practical Departments

**Published:** 1894-04-14

**Authors:** 


### PRACTICAL DEPARTMENTS.

### MODERN BEDSTEADS.

An enormous revolution in the matter of bedsteads and mattresses has been effected of late years, to the manifest advantage of all concerned. Modern bedsteads are as light in construction and form as those of not so very long ago were clumsy and heavy, and the galvanised spring steel wire mattresses which now obtain so universally afford no harbour for dust, and are easily cleansed and disinfected.

The Longford Wire, Iron, and Steel Company (Limited), of Warrington, have achieved a deservedly high reputation in this line, and their bedsteads and "Wood's Patent Wire Mattresses" are to be found in most modern hospitals, and are hardly to be improved upon for comfort and cleanliness. We have received a new catalogue from this firm, which really well repays a careful study. Beds therein shown are adapted for hospital and private use, for invalids and children, and some are specially designed for use in hot climates, provided with appliance for mosquito curtains, &c.

We note bedsteads particularly designed for epileptic patients, with moveable sidepieces; also some of the folding bedsteads, suitable for travelling, are very good, being light and easily portable. Of designs for hospital use there are many. The wire mattresses are guaranteed to bear great weight, and their unstretchable qualities have already received universal acknowledgment.

Castors are an important feature, and these have every attention paid to them. Wood's rubber-covered noiseless castors do not mark floor or carpet, and they allow of bedstead and patient being moved with absolute ease. They are, too, thoroughly strong. Where castors are objected to "Wood's India-rubber Cup Foot" is good, and will give sure satisfaction, this also preventing any marking of the carpet.

### AN IMPROVED GUIDE FOR BATH CHAIRS.

Anyone who has experienced the difficulty of successfully guiding a helpless and possibly heavy invalid in an ordinary bath chair—we allude, of course, to those which are sufficiently light to admit of being pushed by the nurse—will welcome an improvement which will allow the guiding to be accomplished from behind. A method for obtaining this desirable end has been invented by Mr. Septimus Lalonde. This is a wooden handle, which is connected with the front or guiding wheel by chains, and by a slight turning in one direction or the other, the wheel is influenced as desired. This arrangement leaves the hands of the occupant of the chair quite free, and also enables the turning process to be accomplished in a smaller space than by means of a guide-rod used by the invalid. The cost of fitting the appliance is not large, and its adoption should be really a boon both to invalid and attendant.

### BATHS FOR HELPLESS PATIENTS.

A short time since in a medical contemporary Dr. Caldwell Stephen gave a description of a method he has found valuable in the administration of cold baths to typhoid patients.

In cases where this treatment is ordered much difficulty is experienced in lifting the patient in and out of bed and bath, the process often necessitating the assistance of two or three persons, and then being only accomplished with some discomfort to the invalid and anxious effort on the part of the attendants. The number of baths thus given is sometimes very considerable, and the value of the apparatus described by Dr. Stephen will then be abundantly proved.

The plan adopted is to have knocked together by a carpenter two pair of "shears," consisting of two strong wooden beams, some few feet apart at the base and crossed near the top to support a transverse beam. The shears are firmly tied to the foot and head of the bed. "Then I attached," says Dr. Stephen "—to use a sailor's expression—a 'luff tackle purchase' to either end of the cross beam, and from the lower block of each purchase hooked the end of a common Indian hammock. By placing a sheet over the hammock and spreading both out flat over the bed, the patient was able to roll on to it without inconvenience."

By this combination of pulleys the patient can be easily raised, then, while suspended, pulled over the side of the bed and gently lowered into the bath. The return to bed is equally easy. The hammock containing patient and sheet when raised out of the water, should be allowed a second or two for superfluous water to drip off into the bath, then brought to the level of the bed, and pushed on to a rubber sheet. Rubbing dry follows, and the patient is gently rolled on to the further side of the bed, macintosh and sheet being removed.

The whole apparatus is so simple that it can be put together at small cost and in a very short time, and nurses who have to give these baths to heavy and helpless patients will have every reason to be thankful for so materially useful a suggestion, and one, too, which is so practicably obtainable.

